# Recurrent Urethral Phyllodes Tumour Presenting With Urinary Retention: A Case Report and Literature Review

**DOI:** 10.1155/criu/2134277

**Published:** 2026-05-31

**Authors:** M. Sharif, B. Lamb, N. Etessami, L. Lazzereschi, A. Freeman, G. Ellis

**Affiliations:** ^1^ Department of Urology, Royal London Hospital, Barts Health NHS Trust, London, UK, bartshealth.nhs.uk; ^2^ Bart’s Cancer Institute, Queen Mary University of London, London, UK, qmul.ac.uk; ^3^ Department of Histopathology, Royal Free Hospital, Royal Free London NHS Foundation Trust, London, UK, nhs.uk; ^4^ Department of Histopathology, University College London Hospital, University College London Hospitals NHS Foundation Trust, London, UK, nhs.uk; ^5^ Department of Urology, Royal Free Hospital, Royal Free London NHS Foundation Trust, London, UK, nhs.uk

**Keywords:** case report, fibroepithelial polyp, prostatic urethra, recurrence, transurethral resection, urethral phyllodes tumour

## Abstract

We report a rare case of a urethral phyllodes tumour. A 58‐year‐old male presented with urinary retention requiring catheterisation, preceded by a gradual onset of poor urinary flow. He had a prior history of poor urinary flow in 2022, when a urethral fibroepithelial polyp was identified and excised. Histopathology at the time confirmed a fibroepithelial polyp. On recurrence, flexible cystoscopy showed a polyp arising from the prostatic urethra and extending into the bladder neck, while MRI prostate revealed a 63 cc gland with a small median lobe and a PI‐RADS 1, Likert 1 lesion. The patient underwent redo transurethral resection of the lesion, and histology revealed a low‐grade phyllodes tumour. This case underscores the importance of recognising rare urological tumours in order to guide appropriate management.

## 1. Introduction

Fibroepithelial polyps are benign lesions that can occasionally recur and, in rare cases, transform into phyllodes tumour, a low‐grade neoplasm. Their occurrence in the male genital tract is very rare. A detailed understanding of clinical, radiological and pathological features is therefore important to correctly diagnose and guide management.

Phyllodes tumours are very rare fibroepithelial neoplasms, which are characterised by a biphasic pattern consisting of epithelial as well as stromal proliferation. Histologically, they normally show a leaf‐like (phyllodes) architecture where the stromal component can show variable degree of cellularity where proliferation of the epithelial component can also be seen. Depending on the degree of cellularity, atypia, the number of mitotic activity, necrosis and their infiltrative patterns of growth, these lesions can be classified into benign, borderline or malignant.

The breast is the most common site of phyllodes tumours; however, rarely, they have been reported in another anatomical locations such as the pancreas, prostate, male and female genital tract and rarely in the urinary tract, such as the urethra [1].

There are very few reported cases of prostatic/urethral phyllodes tumour, with little or no follow‐up documented.

We report a unique case of a phyllodes tumour, with the patients consent, arising from a recurrent fibroepithelial polyp in the prostatic urethra, providing an insight into the occurrence and management of this rare pathology.

## 2. Case Report

A 58‐year‐old gentleman initially presented in July 2022 with several weeks of an intermittent urinary flow with start–stop pattern. No haematuria, infection or incontinence was reported. The only medical history was of a varicocele treatment 12 years previously. Digital rectal examination suggested an enlarged but benign feeling prostate, and a prostate‐specific antigen (PSA) was normal for age.

**Figure 1:**
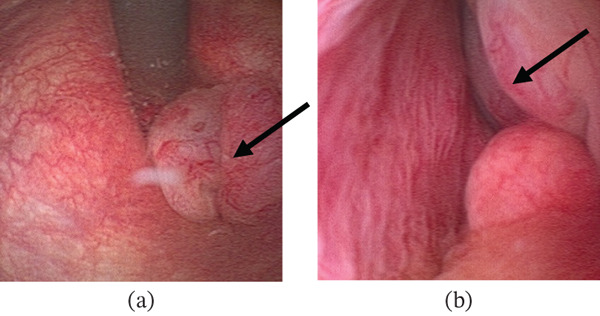
Flexible cystoscopy showing a polyp arising from left side of the distal prostatic urethra extending up to the level of the bladder neck. Image (a) on J manoeuvre and Image (b) within prostatic urethra, both marked with an arrow.

The patient underwent a flexible cystoscopy under local anaesthesia, which revealed a polyp arising on the left side of the distal prostatic urethra adjacent to the verumontanum and extending up to the level of the bladder neck (Figure 1).

The patient underwent a transurethral resection of the lesion under general anaesthesia. Histology of the tissue revealed it to be a fibroepithelial polyp. He was followed up a month later with much improved voiding symptoms.

The patient re‐presented in April 2025 with acute urinary retention requiring catheterisation. He reported a deteriorating flow over the preceding few months.

**Figure 2:**
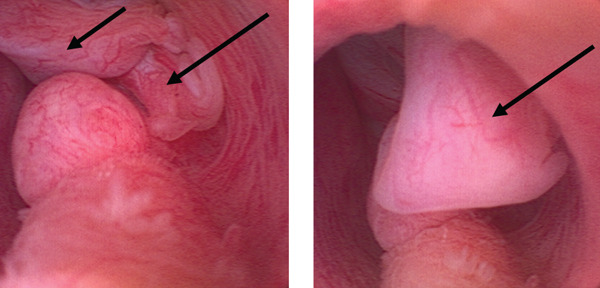
Repeat flexible cystoscopy in 2025, suggesting a recurrent polyp from the same site at the prostatic urethra extending into the bladder neck (marked with arrows).

A flexible cystoscopy was repeated, which demonstrated a recurrent polyp arising from the same site at the prostatic urethra extending into the bladder neck (Figure 2).

**Figure 3:**
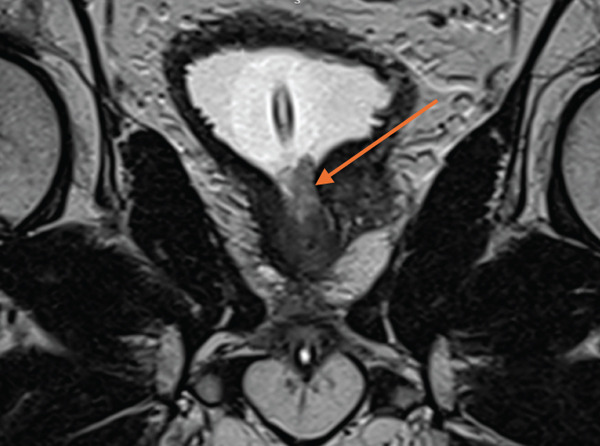
Coronal section of T2‐weighted MRI image showing a non‐enhancing lesion in the prostatic urethra, adjacent to the catheter and extending into the bladder neck, marked with an arrow.

MRI scan of the prostate demonstrated a volume of 63 cc, with the appearance of a small median lobe, with an overall score of Likert 1, suggesting low risk of prostate cancer. Enhancement was seen at the bladder neck, with a non‐enhancing lesion in the prostatic urethra adjacent to the catheter (Figure 3).

The case was discussed in the local cancer multidisciplinary (MDT) team meeting, which concluded that the appearance suggested local resection of the fibroepithelial polyp.

The patient was offered a transurethral resection of the lesion under general anaesthesia, or a HoLEP (HOlmium Laser Enucleation of the Prostate) procedure to reduce the likelihood of future recurrences. The patient opted for a repeat transurethral resection, which was carried out under general anaesthesia. The procedure revealed a polypoid lesion in the prostatic urethra, arising from a broad stalk on the left side of the median lobe, occupying the entirety of the lumen. An en bloc resection of the lesion with bipolar diathermy loop was undertaken, with careful deep resection to ensure its entire removal. The patient underwent a successful trial without catheter the following day.

**Figure 4:**
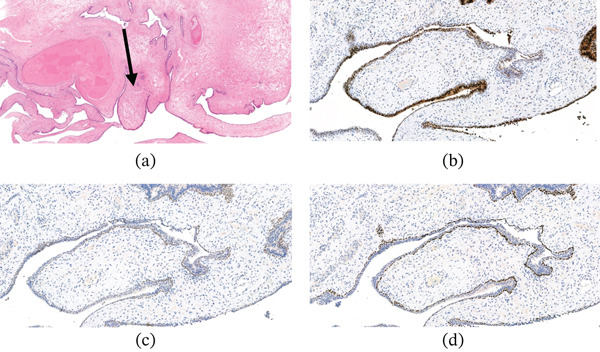
(a) H&E, ×0.4, low power view of the lesion highlighting a polypoid architecture with ‘leaf‐like’ papillary projections(as marked with arrow); (b) NKX3 stain, ×10; (c) GATA3 stain, ×10; (d), p63 stain, ×10.

Histological analysis of the lesion was described as multiple pieces of tissue with a polypoid architecture with a ‘leaf‐like’ papillary projections (Figure 4a).

These are lined by prostatic‐type as well as urothelial‐type epithelial cells, with focal squamous and goblet cell metaplasia.

**Figure 5:**
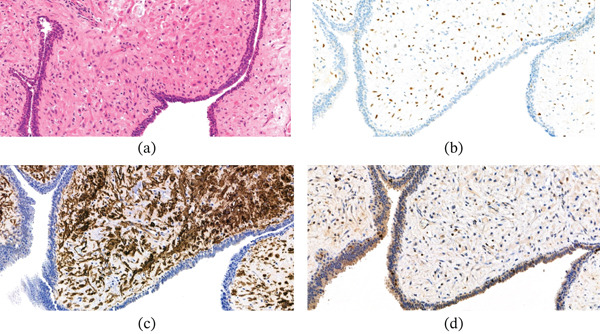
(a) H&E, ×20, high power view of the lesion; (b) ER stain, ×20; (c) SMA stain, ×20; (d), MiB1 proliferation index, ×20, low mitotic activity.

Invagination of the surface epithelium into the stroma is seen, forming cystic structures where the epithelium shows tufting. There is no atypia within the epithelium, which is expressing NKX3 and GATA3 surrounded by P63 basal cells (benign prostatic and urothelial‐type epithelium Figure 4b–d). The stroma is myxoid and it shows different cellularity from poorly cellular areas to slightly more cellular areas. The stroma contains spindled cells, which are expressing SMA (Figure 5c). Caldesmon and Desmin in keeping with their smooth muscle nature. These cells are also expressing ER (Figure 5b). The atypia is minimal and only very occasional mitotic figures are seen (1/50HP) with low proliferation index (Figure 5d). The stroma also contains inflammatory cells including many mast cells. No high‐grade features are seen.

These findings were consistent with low‐grade benign phyllodes tumour of the prostate protruding into the prostatic urethra.

The patient has had complete resolution of symptoms at 6 months of follow‐up. He was given patient initiated follow‐up (PIFU) so they can access help if symptoms recur.

## 3. Discussion

Phyllodes tumour of the prostate is exceedingly rare. They resemble the same tumour when found in the breast, with a biphasic pattern, which comprises of benign glandular epithelium and proliferative, often cellular, stromal component. The stromal characteristics, such as mitotic activity, cellular atypia and nuclear pleomorphism allow it to be classified as low, intermediate or high grade [2].

Fewer than 100 cases of phyllodes tumour of the prostate have been reported in the literature. Bostwick et al. [3] reported 23 cases up until 2002, while Herawi and Epstein [4] review in 2006 suggested 82 cases, which included the Bostwick’s series. Chitale et al. [5] suggested fewer than 100 cases in a time frame up until 2010. All these cases, however, suggested these lesions to be within the prostate rather than a urethral polyp, with identification post‐operatively after a transurethral resection of the prostate.

As Table 1 suggests, there are two documented cases of lesions in the prostatic urethra, with both cases having benign phyllodes tumour and requiring local excision [6, 7].

**Table 1:** Cases in literature on phyllodes tumour of the prostate—In order of anatomical site and citation year [3, 4, 6–12].

Citation (year)	Anatomic site	Number of cases	Grade	Treatment	Follow‐up	Outcome
Agrawal et al., Int Urol Nephrol (2003)	Prostate	1	Malignant phyllodes tumour	Transvesical prostatectomy	Not stated in abstract	Poor prognosis typical of malignant variants
Bostwick et al., J Urol (2004)	Prostate	23	14 low, 7 intermediate, 2 high	TURP/enucleation/prostatectomy (varied)	Up to ≥ 14 years	Recurrence: 50% (low), 86% (intermediate), 100% (high). Metastases in high‐grade; sarcoma emerged in some low/intermediate after multiple recurrences.
Herawi M, Epstein JI. Am J Surg Pathol (2006)	Prostate	50	36 STUMP (varied patterns, incl. phyllodes), 14 stromal sarcomas	Biopsy/TURP/prostatectomy (varied)	Mean 4.9 years	STUMP: some recurred locally, rare progression to sarcoma; sarcomas: high‐grade cases metastasised
Chung et al., Yonsei Med J (2009)	Prostate (large cystic mass)	1	Low‐grade	Tumour excision (patient refused radical prostatectomy)	28 months	Alive, no recurrence/metastasis
Bannowsky et al., J Oncol (2009)	Prostate	1	Low‐grade (‘indolent’)	TURP ×2; surveillance	18 months	Alive, no recurrence
Maudlin et al., BMJ Case Rep (2010)	Prostate (massive, cystic)	1	Not explicitly graded; large phyllodes tumour	LHRH agonist (inoperable)	≈9 months after starting hormonal therapy	Died of renal failure with co‐existent myeloma; lung mass noted (uncertain if metastasis).
Razi et al., Urol Case Rep (2019)	Prostate	1	Low‐grade (benign)	Radical retropubic prostatectomy	12 years	No recurrence
Aydogdu et al., CUAJ (2014)	Prostatic urethra/right lateral lobe (polypoidal lesion)	1	Benign phyllodes tumour	TURP	Not specified; close follow‐up planned	No recurrence reported at time of report
Tang et al., Diagnostic Pathology (2015)	Verumontanum (prostatic urethra)	1	Not graded; phyllodes tumour	Complete local excision	Early postoperative recovery; recommends close follow‐up	Recovered well; no recurrence stated

Patients typically present with obstructive symptoms, generally at a younger age than expected for symptoms linked to benign prostatic hyperplasia (BPH). Presentation with haematuria or palpable mass has also been reported. Some of these tumours have been incidental during BPH surgery.

As in this case, benign tumours are known to have proliferative activity within the prostate without metastatic potential. Those that have the potential to metastasis show greater cellularity, necrosis, stromal overgrowth and mitosis. Common sites of metastasis are the lungs or the bones [13].

Several cases of recurrence of benign phyllodes tumour have been reported; however, these were in patients with incomplete initial resection. In a study following 23 cases over a time frame of 29 years, 50% of low‐grade tumours had recurred, with one patient having metastases. Eighty‐five percent of intermediate tumours had recurrence with one patient having metastasis. All patients with high‐grade tumours had recurrence [3]. In cases with persistent recurrence causing repeated obstructive symptoms, radical prostatectomy may be a viable treatment option, but the known side effects of incontinence and sexual dysfunction that results are well described and may outweigh the intended benefits [14]. Alternative approaches using a combination of repeated cross‐sectional imaging, diagnostic flexible cystoscopy and endoscopic management may be a less invasive method of achieving longer term control, however this does depend on the grading of the tumour. Table 1 summarises the cases, their anatomical location and their follow‐ups.

This case and other cases in the literature highlight that there is no clear understanding of a clear management plan. There are certainly no guidelines; hence, cases should be reviewed in a suitable MDT. The advantages and disadvantages of various surveillance/treatment pathways should be discussed with the patient and documented clearly. This should include the risk of recurrence as well as progression, where applicable. Having access to PIFU, where benign tumours involved, such as this case may help the patient seek medical attention for when required.

## 4. Conclusion

This case highlights the potential for recurrence of benign epithelial polyp and prostatic urethral phyllodes tumour, and our experience of management with imaging and endoscopic resection. It also highlights the importance of careful investigation in cases of atypical lower urinary tract symptoms, so as not to misdiagnose those resulting from benign enlargement of the prostate alone. Follow‐up for patients can vary depending on the outcome, as suggested above.

## Author Contributions

**M. Sharif:** conception of the work, data collection, literature review, and drafting of the manuscript. **B. Lamb and G. Ellis:** supervision, critical revision of the manuscript, and overall oversight. **N. Etessami, L. Lazzereschi, A. Freeman:** histopathological analysis and contribution to manuscript revision.

## Funding

No funding was received for this manuscript.

## Disclosure

All authors have read and approved the final version of the manuscript. Mr. B Lamb had full access to all of the data in this study and takes complete responsibility for the integrity of the data and the accuracy of the data analysis.

## Consent

All the patients allowed personal data processing and informed consent was obtained from all individual participants included in the study.

## Conflicts of Interest

The authors declare no conflicts of interest.

## Data Availability

The data supporting the findings of this case report are available from the corresponding author upon reasonable request, subject to patient confidentiality considerations.
